# Online Cognitive-Behavioral Therapy-Based Nutritional Intervention via Instagram for Overweight and Obesity

**DOI:** 10.3390/nu16234045

**Published:** 2024-11-26

**Authors:** Greta Gabriela Rychescki, Gabriela Rocha dos Santos, Caroline Fedozzi Bertin, Clara Nogueira Pacheco, Luciana da Conceição Antunes, Fatima Cody Stanford, Brunna Boaventura

**Affiliations:** 1 Department of Nutrition, Health Sciences Center, Universidade Federal de Santa Catarina, Florianópolis 88040-370, SC, Brazil; 2 Graduate Program in Psychiatry and Behavioral Sciences, Universidade Federal do Rio Grande do Sul, Porto Alegre 90035-003, RS, Brazil; 3 MGH Weight Center, Massachusetts General Hospital, Boston, MA 02114, USA; 4 Harvard Medical School, Boston, MA 02115, USA; 5 Nutrition Obesity Research Center, Boston, MA 02114, USA; 6 Department of Medicine-Neuroendocrine Unit and Department of Pediatrics-Endocrinology, Massachusetts General Hospital, Boston, MA 02114, USA

**Keywords:** internet-based intervention, obesity, eating behavior, lifestyle modification, social media, online protocol

## Abstract

Background/Objectives: Obesity is a multifactorial chronic disease treated through lifestyle modifications, pharmacotherapy, and surgery. With the rise of social media, platforms like Instagram have become tools for lifestyle interventions. This study evaluated the impact of a cognitive-behavioral-therapy-based nutritional intervention via Instagram on body weight, eating behavior, and mental health in individuals with overweight and obesity. Methods: A 5-week online intervention delivered daily nutritional, cognitive, and behavioral content via a private Instagram account using live sessions, reels, feed posts, polls, and stories. Standardized dietary plans were sent by e-mail. Self-reported weight and waist circumference and questionnaires on eating behavior, self-esteem, stress, and anxiety were collected. Engagement and interaction were measured through comments, likes, number of followers, story retention, participation in live sessions, and direct messages. Results: The final sample included 66 participants (63 women), 27 with overweight and 39 with obesity, and a mean age of 40.5 ± 10.6 years. After the intervention, body weight decreased by 1.1 kg, while waist circumference remained unchanged. Participants with obesity showed significant improvements in binge eating, uncontrolled eating, self-esteem, stress, and anxiety, while those with overweight showed improvements in binge eating and stress. Weight loss was associated with reduced binge eating and lower cognitive restriction, while lower uncontrolled eating was related to decreased emotional eating, anxiety, and stress. Additionally, participation in live sessions was associated with reduced binge eating. Conclusions: This online intervention via Instagram was effective in improving weight loss, eating behavior, and mental health symptoms in participants with overweight and obesity.

## 1. Introduction

By 2035, estimates for global levels of overweight and obesity suggest that nearly 3.3 billion adults may be affected, compared to 2.2 billion in 2020, reflecting an increase from 42% of adults in 2020 to over 54% by 2035 [1]. Obesity is recognized as a multifactorial chronic disease, with causes that include genetic, environmental, social, and behavioral factors [2,3,4]. Lifestyle interventions, including nutritional therapies with or without exercise, form the cornerstone of obesity management and remain crucial even when additional treatments, such as pharmacotherapy or surgery, are needed to manage the disease effectively [4].

Although lifestyle modification is essential for weight reduction, it often faces challenges that prevent long-term weight regain, primarily due to patients’ difficulty adhering to these behavioral changes [5,6]. In this context, cognitive behavioral therapy (CBT) is widely used in weight loss counseling and promotes lasting changes through strategies such as goal setting, problem-solving, stimulus control, and self-monitoring [7,8,9]. Psychological mechanisms involved in CBT, such as resilience and self-efficacy, play a crucial role in sustaining long-term behavioral changes, especially in eating behaviors [10].

Given this, it is vital to embrace innovative, low-cost strategies that extend beyond traditional diet interventions, integrating proven methods like CBT to enhance accessibility and impact. By aligning with modern social behaviors and fostering diversity, equity, and inclusion, these approaches can break down barriers, reduce stigma and weight bias, and empower individuals with effective, practical, and compassionate care.

In recent years, social media has emerged as a powerful tool for communication and social influence [11]. Instagram has shown promise in health promotion, serving as an online communication channel that facilitates community interaction and content sharing [12]. Studies indicate that social media interventions improve engagement by up to 82.9% and positively impact health behaviors by 88.8%, reaching various age groups and cultural backgrounds [13,14,15,16].

This study is pioneering in using Instagram for CBT-based nutritional intervention aimed at individuals with overweight and obesity. The hypotheses of this study are as follows: (1) the intervention will result in significant reductions in weight, improvements in mental health parameters, and reductions in dysfunctional eating behaviors among individuals with overweight and obesity; (2) active engagement in live sessions and Instagram interactions may amplify these effects. The primary research questions are as follows: how does this Instagram-mediated intervention affect participants’ body weight, eating behavior, and mental health parameters? To what extent does engagement on the platform influence these effects?

## 2. Materials and Methods

### 2.1. Study Design and Participants

**Figure 1:**
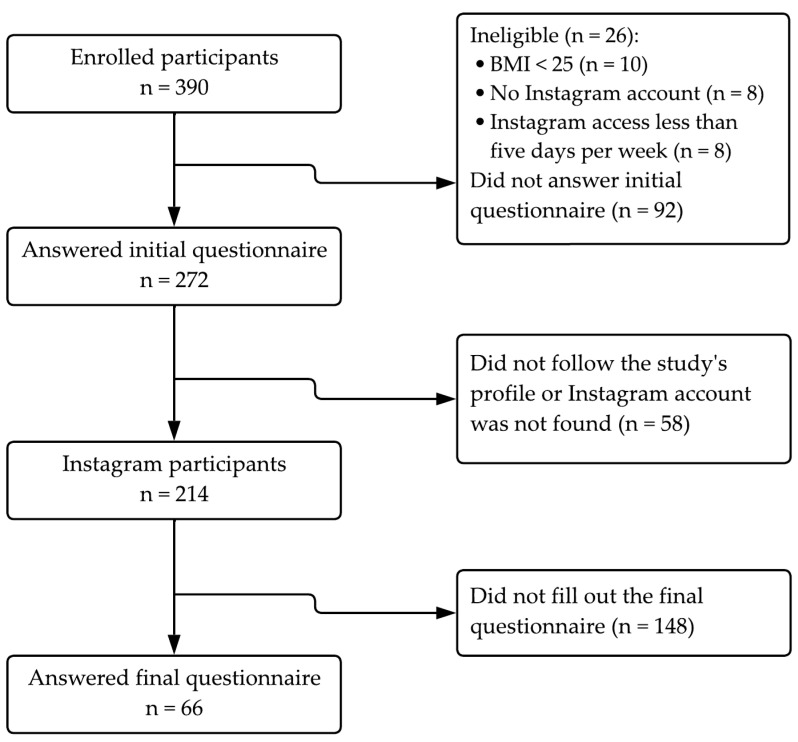
Flowchart of participants throughout the intervention study.

This study was conducted as a quantitative and experimental trial. It was performed under the ethical principles outlined in the Declaration of Helsinki for Medical Research involving human subjects. It was approved by the Ethics Committee for Research with Human Beings at the Federal University of Santa Catarina (CAAE 63286322.4.0000.0121). The sample was recruited by convenience in October 2022, and recruitment was carried out through social media accounts of research collaborators, partner research projects, and the university’s newsletter. The screening, eligibility criteria, and analyzed data are reported in Figure 1. The inclusion criteria were individuals over 18 years of age with a body mass index (BMI) ≥ 25 kg/m^2^, an active Instagram account, internet access via smartphone, and access to Instagram a minimum of five days per week.

### 2.2. Data Collection

A Google Forms questionnaire, which included the Informed Consent Form, was used for registration. At the beginning and end of the intervention, participants completed a questionnaire that included socio-demographic questions and self-reported weight, height, and waist circumference. Validated questionnaires to measure eating behavior and mental health were included: the Rosenberg Self-Esteem Scale (RSE) [17]; the Binge Eating Scale (BES) [18]; the 21-item Three-Factor Eating Questionnaire [TFEQ-R21] [19]; the State-Trait Anxiety Inventory (STAI) [20]; and the Perceived Stress Scale (PSS) [21].

The RSE, developed in 1989 and used worldwide to assess self-esteem in the community [22], was revalidated in Brazil in 2011 [17]. This scale consists of 10 items with positive and negative statements to evaluate an individual’s ability to self-appraise. Respondents indicate their level of agreement on a scale ranging from 1 (strongly disagree) to 4 (strongly agree).

BES is a self-report questionnaire designed for use in individuals with obesity, which is intended to assess the presence of binge eating characteristics indicative of an eating disorder and primarily serves as a screening tool [18]. It analyzes two components of binge eating: behavioral manifestations (e.g., eating quickly, overeating) and the emotions/cognitions that precede or follow binge eating episodes (e.g., feeling out of control, guilt) [23]. Scores on the BES range from 0 to 46, with moderate and severe binge eating corresponding to cutoff scores of 18 and 27, respectively.

Eating behavior was evaluated using the TFEQ-R21, developed to assess cognitive restraint, disinhibition, and hunger in individuals with obesity [19,24]. The TFEQ-R21 covers three domains of eating behavior: cognitive restraint, which evaluates control over food intake to influence body weight and shape; emotional eating, which measures the tendency to overeat in response to negative mood states; and uncontrolled eating, which assesses the tendency to lose control overeating when feeling hungry or when exposed to external stimuli. The response format includes a 4-point scale for items 1 to 20 on the TFEQ-R21 and a numerical rating scale for item 21. Higher scores indicate greater cognitive restraint, uncontrolled eating, or emotional eating.

The STAI includes a scale that measures anxiety as a state (STAI-S) and another that assesses anxiety as a trait (STAI-T) in adults [20,25]. According to this inventory, the state scale requires participants to describe how they feel “now, at this moment”, while the trait scale asks how individuals “generally feel”. Both subscales consist of 20 items rated on a Likert scale with four response options (0 to 3). The scores for both scales are obtained by summing the item responses.

The PSS is a well-established tool that measures the subjective report of stressful situations over the past month [21]. It consists of 14 questions with response options ranging from 0 to 4. The total score is the sum of the responses to these 14 questions, with scores varying from 0 to 56.

Different engagement data were also collected, as the intervention was conducted through a social network [26]. The total number of comments and likes on feed posts from each participant was used to assess individual engagement. Additionally, interactions of participants who completed the intervention were evaluated for their participation in live sessions and direct messages. The Public Engagement Rate (PER) [Public Engagement Rate = (likes + comments/number of followers) × 100] was calculated based on the number of followers. The Stories Retention Rate (SRR) [SRR = (views of the last story/views of the first story) × 100] was used to calculate the percentage of followers who watched the story sequences to the end on the same day [27,28]. In the calculation of PER and SRR rates, the total number of followers of the study’s Instagram account was considered (*n* = 214). Although Instagram provides data analysis tools for professional accounts through Instagram Business via Facebook, private accounts need access to these automated tools, requiring all data collection to be conducted manually.

### 2.3. Intervention Protocol

The intervention protocol, developed in Brazilian Portuguese, lasted five weeks, with each week centered on a specific theme. The intervention was delivered through a private Instagram account (@emagrece.com), managed and moderated by the researchers.

The protocol included a variety of resources offered by the platform, such as feed posts, reels, stories, and live sessions, with weekly live classes hosted by experts. The protocol concluded with 584 posts (54 image posts, 8 videos/lives, 522 stories), and educational materials were made available in the bio, such as e-books. Additionally, dietary plans were provided to participants via email. Four dietary plans (1500 kcal, 1800 kcal, 2200 kcal, and 2500 kcal) were developed, and each participant received the one closest to their energy needs [29].

Given that this protocol is a pioneering use of this social media platform, its development was primarily based on references from CBT, and specific protocols within this approach focused on weight loss [6,30,31,32]. In the book *The Diet Trap Solution: Train Your Brain to Lose Weight and Keep It Off for Good*, Beck and Busis make CBT accessible to the public by using clear language centered on everyday difficulties and presenting practical strategies [33]. This book served as an essential reference for translating theory into practices that the participants could easily understand and apply.

As a pre-protocol, three introductory posts were made to explain the intervention, introduce the researchers, and cover content related to the stages of habit change, utilizing the transtheoretical model [34]. The purpose of this content was to prepare participants for the behavioral changes they would be encouraged to adopt throughout the intervention, prompting them to reflect on their current stage of readiness for change and whether they wanted to remain in that stage or move beyond ambivalence.

The first week, titled “Fundamental Strategies”, focused on building a solid foundation for long-term weight loss by exploring skills, strategies, habits, and goals, themes present in CBT-based treatments [6,30,32]. The “Advantages List” was highlighted as an essential motivational tool, as it addresses cognitive aspects and reminds individuals of their goals in response to food temptations, being a strategy for implementing intentions and episodic future thinking [33,35,36]. Additionally, techniques such as self-praise and recording achievements were introduced to strengthen participants’ motivation and resilience, given that individuals often experience internalized weight bias, which can negatively influence their self-evaluation and information processing [30].

In the second week, it was focused on “Eating and Emotions”. Strategies were discussed for dealing with emotional eating, psychological traps, and dichotomous thinking patterns [6,32]. The cognitive continuum technique was used to seek flexibility in beliefs that reflect polarized thinking, both intellectually and emotionally [30]. Additionally, methods for managing hunger and food cravings were presented, such as creating a distraction list [30]. Individuals had their first contact with the concept of automatic and dysfunctional thoughts and how to deal with them by creating adaptive responses and alternative behaviors in response to a trigger [30,35].

The third week’s theme was “Preparing Healthy Meals”, emphasizing the importance of proper nutrition and balanced meal composition, as the CBT approach for weight loss also considers dietary recommendations [6,32]. Practical guidance was provided on selecting and combining foods to optimize meal quality and quantity. Adopting mindful eating practices was also encouraged, as this approach has been demonstrated to promote healthier eating behaviors, probably by increasing awareness of both internal and external cues related to eating [37].

In the fourth week, “Organization and Planning” was addressed, highlighting the importance of self-care, personal development, organization, and culinary skills in maintaining a healthy diet. The individuals were exposed to the need to make weight loss one of their priorities and actively change their habits and routines [32]. Moreover, strategies for meal planning, preparation, and organizing the food environment were discussed to facilitate adherence to the dietary plan and promote participants’ autonomy [6].

Finally, the fifth week explored “Dysfunctional Thoughts and Routine Disruptions”. Automatic and confrontational thoughts continued to be explored to help develop flexible eating strategies, often more manageable and effective than rigid dietary rules. By fostering adaptability, this approach reduces the risk of protocol abandonment and subsequent weight regain [32]. Strategies were discussed for managing cognitive distortions, dysfunctional eating behaviors, and challenging situations, particularly in the context of festivities and social events where food temptations are more frequent [33].

The topics covered in the posts and the scripts for the weekly live sessions were previously structured and are available in the Supplementary Materials.

The intervention was conducted through a private Instagram account, with participants manually accepted by the research team after verification of their initial questionnaire responses. This process eliminated the need for CAPTCHA (Completely Automated Public Turing Test to Tell Computers and Humans Apart) to filter out automated accounts, ensuring all engagement data came from verified human participants.

### 2.4. Statistical Analysis

Data are presented as means and standard deviations. Statistical analysis was conducted using the Shapiro–Wilk test to assess normality. The independent samples *t*-test was used to evaluate differences in baseline characteristics between the overweight and obesity groups, while the paired Student’s *t*-test was employed to compare pre- and post-intervention periods. The SPSS Statistics program was used for statistical analysis, with a significance level of 5% being adopted. Additionally, the effect size was calculated to provide a standardized measure of the magnitude and practical significance of the observed differences, offering insight beyond statistical significance. Cohen’s *d* value was interpreted as indicative of trivial (<0.20), small (0.20 to 0.49), medium (0.50 to 0.79), and large (≥0.8) effect sizes [38]. Pearson’s correlation analysis was used to assess the association between the parameters evaluated before and after the intervention and Instagram engagement and interaction measures, with results from participants with overweight and obesity combined.

## 3. Results

Table 1 presents the characteristics of the participants that completed the intervention (*n* = 66), which were predominantly female (*n* = 63; 95.4%), with 60.6% of the participants aged between 31 and 50 years (*n* = 40). Additionally, according to their BMI, 59.1% of the participants had obesity (*n* = 39), and 40.9% had overweight (*n* = 27). The majority of the sample had a high level of education, with 92.4% holding postgraduate degrees (*n* = 46). A prevalence of anxiety was observed among the participants (*n* = 59), along with metabolic diseases (*n* = 38), including diabetes mellitus, dyslipidemia, and hypertension.

Table 2 presents the baseline characteristics and the results of a 5-week online intervention in individuals with overweight and obesity, showing significant improvements across several parameters related to weight, eating behavior, and mental health. Notably, the outcomes were more pronounced in the group with obesity compared to the overweight group. It is noteworthy that in the baseline comparison between groups, significant differences were observed in weight, waist circumference, binge eating, and anxiety, as shown in Table 2.

After the intervention, a significant reduction in body weight was observed for both participants with overweight (*p* = 0.002) and obesity (*p* = 0.01), with medium (*d* = 0.601) and small (*d* = 0.392) effect sizes, respectively. However, neither group observed any significant changes in waist circumference, suggesting that the weight loss may not have been primarily in the abdominal region.

Regarding eating behavior, binge eating improved significantly in both groups (*p* < 0.001), showing a medium effect size. Uncontrolled eating showed a significant improvement only in the group with obesity (*p* = 0.009), with a small effect size (*d* = 0.398).

As for mental health, there was a significant increase in self-esteem only in the obesity group (*p* = 0.027), with a small effect size (*d* = 0.319). Additionally, anxiety decreased significantly only in the obesity group (*p* = 0.001), with a medium effect size (*d* = 0.529). Perceived stress significantly reduced in both groups, with a small and medium effect size in obesity (*p* = 0.045; *d* = 0.279) and overweight (*p* ≤ 0.001; *d* = 0.698), respectively.

Some significant correlations were also observed, as shown in Table 3. A reduction in body weight was positively correlated with a decrease in binge eating behavior and inversely correlated with cognitive restriction. A reduction in uncontrolled eating was associated with reduced emotional eating, anxiety, and stress levels. Lower stress levels were also linked to a further decrease in anxiety.

**Figure 2:**
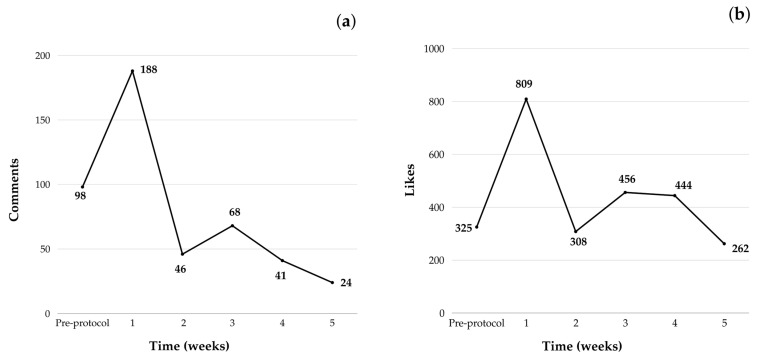

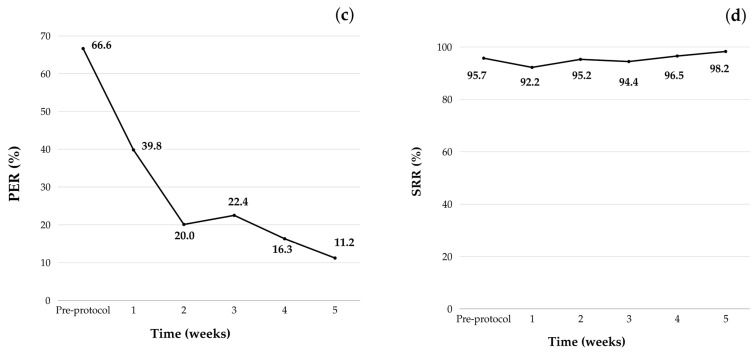
Engagement metrics per week of the study (*n* = 214). (**a**) Average number of comments on the feed posts; (**b**) average number of likes on the feed posts; (**c**) average Public Engagement Rate (PER); (**d**) average Stories Retention Rate (SRR).

Figure 2 presents engagement metrics, including the average number of likes and comments per week, along with the PER and SRR for all followers of the study’s Instagram account (*n* = 214), not just those who completed the intervention protocol (*n* = 66). A decline in the number of comments and likes on the posts was observed throughout the intervention weeks, particularly compared to the engagement levels in the first week. A similar trend was seen with the PER, indicating a general decrease in engagement throughout the protocol. However, the SRR remained stable, suggesting that participants in the study continued to engage with the stories, maintaining consistent interest in the content over time. In the correlation analysis (Table 3), engagement, measured by the total number of comments and likes, along with interactions such as participation in live sessions and direct messages, showed no significant correlations with changes in body weight or mental health outcomes among participants who completed the intervention. However, higher attendance in live sessions was associated with a reduced tendency toward binge eating. Additionally, higher engagement on Instagram (comments and likes) was correlated with increased live session participation and more frequent communication through direct messages.

## 4. Discussion

Although several studies have explored the use of social media for nutritional protocols, this is the first to implement a nutritional intervention on Instagram, incorporating CBT strategies for individuals with overweight and obesity [39,40]. This study was guided by two primary hypotheses: (1) that the intervention would lead to significant reductions in weight, improvements in mental health, and reductions in dysfunctional eating behaviors among individuals with overweight and obesity, and (2) that active engagement in live sessions and interactions on Instagram would further enhance these effects. In response to the first hypothesis, the results showed weight reduction, improvements in mental health parameters, and a decrease in binge eating and uncontrolled eating, particularly within the obesity group. Considering hypothesis 2, a reduction in binge eating was associated with greater participation in live sessions. However, other engagement and interaction parameters with the platform did not demonstrate additional benefits for the other evaluated outcomes.

Regarding the primary outcome, this study found a reduction in participants’ body weight, with an average loss of 1.1 kg over the 5-week intervention. This finding is consistent with other studies that utilize social media-based interventions and CBT approaches for weight management [26,39,40]. The modest weight loss observed may be explained by the short duration of the intervention and its self-directed nature.

This study found a significant reduction in binge eating symptoms, with a medium effect size, shifting the classification from moderate to possibly absent. While the questionnaire used assesses symptoms rather than diagnosing binge eating disorder (BED), this outcome is encouraging, as BED is linked to various functional impairments, including reduced social performance, diminished quality of life, and increased morbidity [41]. The improvement can be attributed to the use of CBT strategies, which are considered the gold standard for treating BED, as well as mindful eating practices that foster greater awareness and are associated with reductions in anxiety, depression, and binge eating episodes [42,43,44]. These results are promising given the association between BED and a higher risk of weight gain and obesity development [40].

In terms of eating behavior, significant improvements were observed in uncontrolled eating among participants with obesity, while no changes were noted in emotional eating or cognitive restraint. Although all domains were expected to improve due to the CBT strategies employed, previous studies have also reported mixed and sometimes contradictory results [40,45,46]. Reducing uncontrolled eating suggests better dietary control in response to external stimuli, a critical factor in maintaining body weight and ensuring the long-term success of obesity treatment. Uncontrolled eating is a crucial domain in this process [47,48,49].

The findings of this study showed a reduction in anxiety levels among participants with obesity and a decrease in perceived stress in both participants with overweight and obesity after the intervention. These results align with previous research highlighting the effectiveness of psychosocial interventions in enhancing the emotional well-being of individuals with obesity [50,51]. While modest, these reductions are significant given that individuals with obesity often face heightened anxiety and stress due to the social stigma associated with excess weight [52].

Some parameters analyzed showed significant improvement only in the obesity group and not the overweight group. Baseline differences in psychological and physiological characteristics, such as weight, waist circumference, binge eating symptoms, and anxiety, between the two groups may have contributed to variations in how participants from each group responded to the intervention. Individuals with obesity may face more severe or complex psychological challenges, such as issues related to eating behavior, self-perception, and anxiety [53]. These challenges, combined with the cognitive and emotional burden associated with obesity, could affect their level of engagement and responsiveness to behavior change strategies, ultimately resulting in different outcomes compared to those with overweight [53]. In fact, the relationship between obesity, eating behavior, and mental health is complex and interconnected. Research suggests that weight stigma can intensify feelings of anxiety and stress, creating a vicious cycle that impedes weight loss and the adoption of healthy habits [54]. By reducing stress and anxiety, the intervention may have contributed to improved self-esteem in participants with obesity. Self-esteem is a vital component of mental health and quality of life, and individuals with obesity often struggle in this area due to discrimination and prejudice [55,56].

Furthermore, weight stigma has significant implications for both the mental and physical health of individuals with obesity. Those who experience weight stigmatization are at higher risk of developing eating disorders, depression, and anxiety [52]. By reducing anxiety and stress, the intervention may have created a more supportive environment for participants to better cope with weight stigma and develop a more positive body image. Although body shame and internalized stigma were not specifically assessed, reducing these factors can enhance self-esteem, self-efficacy, and self-confidence, encouraging greater participation in weight loss programs and social activities and ultimately improving overall well-being [57]. This underscores the importance of holistic obesity treatment approaches that address the physical, emotional, and psychological dimensions of health [58].

This study highlights the significant potential of using Instagram to deliver a health intervention protocol, leveraging digital strategies to maximize participant engagement, as reflected in their involvement. A notable feature of this study is the sample’s age range, with the majority (60.6%) between 31 and 50 years old, which differs from most related research that typically focuses on participants under 30 [26]. This suggests that Instagram’s reach is open to more than just young adults but can be effectively utilized with a more diverse audience. However, most of the sample consisted of women with postgraduate degrees, a factor that may have influenced engagement and results.

Digital media engagement in health interventions is a relatively new area of research, with limited research available. Although no definition exists, engagement is often associated with cognitive, emotional, and behavioral involvement [59,60]. It reflects the audience’s reactions to social media content, including their feelings and actions, such as sharing, searching, and commenting [61].

The total number of comments and likes on feed posts for each participant who completed the intervention was not associated with weight loss or the other improvements observed in the study. Comments and likes on the feed indicated that engagement decreased over the intervention, with the highest levels observed in the first week. This decline may suggest participant dropout and reduced content interaction over time. This pattern aligns with findings from obesity treatment studies, where participants often begin with high expectations but gradually lose engagement [62].

Participant dropout may have significantly impacted overall engagement levels, as 214 participants initially enrolled in the intervention, but only 66 completed the final assessments. This attrition likely influenced the decline in PER values, calculated based on the number of comments and likes divided by the total number of followers rather than solely on those who completed the intervention. Despite the decrease in PER from 39.8% in the first week of the intervention to 11.2% in the final week, the ending value still surpassed the reference level of 10% for accounts with fewer than ten thousand followers, indicating strong overall engagement [30]. Considering the delivery format through stories, the SRR indicated that despite declining total views, retention remained consistently high throughout the intervention, averaging 94%, significantly above the typical 75% average for accounts with fewer than ten thousand followers [30]. Story engagement reflects participants’ preference for authentic, real-time content, suggesting that those who remained engaged continued to consume a significant portion of the content provided. Instagram’s story feature enables quicker and more spontaneous communication that fosters a personal and direct connection with the audience, making it one of the most popular formats for attracting followers on Instagram [63,64]. However, an analysis of another type of interaction, participation in live sessions, demonstrated a positive correlation between higher attendance and improvements in binge eating behavior.

Although no association was found between any form of engagement analyzed and weight loss, different types of engagement or interactions may be linked to distinct objectives and outcomes. Notably, despite the modest weight loss observed, eating behavior and mental health aspects significantly improved with the intervention protocol. This aligns with a health-focused approach, rather than solely emphasizing weight loss, which should be considered when treating overweight and obesity [65]. In this context, integrating diverse Instagram content delivery formats with dietary guidance and CBT presents a potentially effective strategy for further investigating interventions targeting overweight and obesity treatments.

The strengths of this study include the use of an innovative, science-based intervention protocol delivered through a contemporary social media platform that is widely utilized by the general population. Nutritional intervention studies for the management of overweight and obesity must align with current population behaviors, offering intervention models adapted to people’s needs and interests. Moreover, it is crucial to ensure that information in such interventions is grounded in robust scientific evidence, such as CBT for managing behavioral modification of overweight and obesity. Solely providing dietary information or a meal plan is often insufficient, as many individuals struggle with behavioral aspects, dysfunctional thoughts, and difficulties integrating changes into their daily routines. Another strength of this intervention was the high frequency of contact with participants, occurring daily and multiple times per day. Frequent engagement is positively associated with sustainable lifestyle changes. In a conventional setting, interactions would be far more sporadic and incur significantly higher costs. Additionally, most participants were in the 31- to 50-year-old age range, a demographic group typically underrepresented in studies involving digital platforms or social media interventions. Furthermore, as Instagram offers a variety of content delivery formats, this study utilized a diverse range of methodologies, including videos, images, easy-to-understand written content, live sessions, polls, and messaging for interacting and answering questions. In particular, the use of stories allowed for topics typically discussed during in-person or telehealth consultations to be addressed, fostering interaction through questions and answers for the entire intervention group. This comprehensive approach allowed for a more tailored and engaging experience, catering to participants’ diverse preferences and needs. Finally, another strength of this study was the use of a stigma-free intervention environment, as many individuals with obesity distance themselves from healthcare settings and professionals due to feelings of shame, guilt, fear of discrimination, or inadequately accommodating physical settings. A digital interface allows patients to engage without fear of judgment, progress at their own pace, and gradually incorporate changes. This study provides a novel contribution to the field of health interventions by offering a non-conventional, low-cost, scalable approach that yields interesting and applicable results, potentially becoming more common and accessible in the future.

Despite the results observed, this study has several limitations. First, the 5-week duration restricted the evaluation to short-term outcomes, with no follow-up to assess long-term effects. Additionally, participant attrition occurred during the selection process and the intervention, reducing the final sample size. Since the protocol was conducted online, participant engagement relied heavily on their motivation to comment on posts and ask questions. This and the relatively short duration may have contributed to the dropout rate. Despite providing instructional videos demonstrating how to measure waist circumference and detailed guidance on conducting body weight assessments, the use of self-reported anthropometric data introduces the potential for inaccuracies in reporting, potentially affecting the credibility of the results. Furthermore, the absence of a control group means that the results are subject to external factors beyond the researchers’ control, highlighting the need for controlled clinical trials to confirm these findings. Another limitation lies in the demographic composition of the sample, with the majority of participants being women, which may limit the generalizability of the results to men. Additionally, the high level of education among participants may be considered a limitation, as it prevents an assessment of the intervention’s applicability in a sample with fewer years of formal education. Finally, there were challenges in implementing the protocol, as daily posts and interactions on the social network were required to maintain participant engagement and ensure consistent content delivery. Instagram’s algorithm also poses limitations, as it does not consistently deliver all posts and stories to followers. Determining the optimal number of posts and weekly interactions was challenging, as no similar studies were available to guide the design. Therefore, participant attrition during the intervention may have been partly due to low content delivery for some individuals.

## 5. Conclusions

This study demonstrated the effectiveness of using Instagram for cognitive-behavioral therapy-based nutritional intervention in users with overweight and obesity. During the 5-week intervention, participants achieved a modest weight loss of 1.1 kg and improved eating behaviors and mental health, particularly among those with obesity. These findings, from a combined online CBT and nutritional approach, are essential for long-term treatment success, demonstrating mental, physical, and behavioral benefits for individuals with overweight and obesity.

Low-cost, wide-reaching interventions like the one in this study have the potential to be implemented in public health systems, benefiting a larger population of individuals with overweight and obesity, reducing weight stigma, and promoting more significant health equity in the fight against this epidemic. Further research, mainly controlled studies with larger sample sizes and longer durations, must confirm these findings. It would also be valuable to explore this type of intervention in combination with pharmacological treatments and bariatric surgery to assess its effectiveness as part of a comprehensive obesity management strategy.

## Figures and Tables

**Table 1:** Sociodemographic and clinical characteristics of the study participants (*n* = 66).

Variable	Number of Participants (%)	
Age (years)	18 to 30	14 (21.2)
	31 to 50	40 (60.6)
	≥51	12 (18.2)
Gender	Female	63 (95.4)
	Male	3 (4.5)
Education level (years)	≤7	1 (3.0)
	8 to 10	3 (4.5)
	≥11	62 (92.4)
Diseases/Complications	Metabolic Diseases ^1^	38 (57.6)
	Hormonal Issues ^2^	21 (31.8)
	Depression	14 (21.2)
	Anxiety	59 (89.4)
	Insomnia	20 (30.3)
	Cancer	3 (4.5)
	Respiratory Issues ^3^	12 (18.2)
BMI (kg/m^2^)	Overweight (25 to 29.9 kg/m^2^)	27 (40.9)
	Obesity (30 to ≥ 40.0 kg/m^2^)	39 (59.1)

BMI, body mass index. ^1^ Type 2 diabetes mellitus, pre-diabetes, dyslipidemia, and/or hypertension. ^2^ Polycystic ovary syndrome, hypothyroidism, and/or hyperthyroidism. ^3^ Asthma and/or sleep apnea.

**Table 2:** Baseline characteristics and changes in body weight, eating behavior, and mental health parameters after 5 weeks of online intervention in participants with overweight and obesity (*n* = 66).

Parameters	Overweight (*n* = 27)				Obesity (*n* = 39)			
	Basal	5 Weeks	*p*	*d* [IC95]	Basal	5 Weeks	*p*	*d* [IC95]
Body weight (kg)	73.9 ± 7.8	72.8 ± 7.4	0.002 *	0.601 [0.18–1.00]	97.2 ± 15.9 ^†^	96.1 ± 15.2	0.010 *	0.392 [0.06–0.71]
Waist circumference (cm)	84.6 ± 7.8	83.5 ± 9.1	0.080	0.268 [−0.11–0.64]	104.4 ± 10.8 ^†^	102.5 ± 10.9	0.147	0.171 [−0.14–0.48]
Binge eating	16.5 ± 7.5	12.0 ± 6.0	<0.001 *	0.697 [0.27–1.11]	24.4 ± 8.0 ^†^	18.5 ± 9.9	<0.001 *	0.656 [0.30–0.99]
Emotional eating	14.2 ± 4.3	14.9 ± 3.9	0.180	0.179 [−0.55–0.20]	15.4 ± 6.0	14.9 ± 4.7	0.246	0.111 [−0.20–0.42]
Cognitive restriction	15.5 ± 1.9	15.8 ± 2.5	0.343	0.079 [−0.45–0.30]	15.4 ± 3.0	15.5 ± 2.4	0.389	0.045 [−0.35–0.26]
Uncontrolled eating	22.4 ± 4.9	21.8 ± 4.7	0.186	0.175 [−0.20–0.55]	23.8 ± 4.3	22.1 ± 4.3	0.009 *	0.398 [0.06–0.72]
Self-esteem	30.1 ± 4.9	31.7 ± 4.8	0.101	0.252 [−0.63–0.13]	27.8 ± 5.5	29.6 ± 4.8	0.027 *	0.319 [−0.63–0.1]
Anxiety	46.9 ± 9.2	45.7 ± 8.5	0.148	0.206 [−0.17–0.58]	53.6 ± 7.8 ^†^	50.7 ± 7.6	0.001 *	0.529 [0.19–0.86]
Perceived stress	29.1 ± 4.9	23.5 ± 7.5	<0.001 *	0.698 [0.27–1.11]	30.0 ± 7.7	27.7 ± 7.2	0.045 *	0.279 [−0.43–0.59]

Data are presented as mean ± standard deviation. * Significant differences after the intervention within each group compared to baseline values (*p* < 0.05). ^†^ Significant difference between baseline values of the overweight and obesity groups (*p* < 0.05). *d* = Cohen’s *d* effect size (trivial, <0.20; small, 0.20 to 0.49; medium, 0.50 to 0.79; and large, ≥0.8).

**Table 3:** Correlation analysis of body weight, eating behavior, mental health, engagement, and interaction parameters after five weeks of online intervention in participants with overweight and obesity (*n* = 66).

	1	2	3	4	5	6	7	8	9	10	11
1. Body weight	1	**0.318**	0.047	**−0.280**	0.101	−0.143	0.005	0.122	0.046	−0.191	0.041
2. Binge eating	**0.318**	1	−0.188	−0.059	−0.079	−0.075	0.162	0.051	−0.097	**−0.286**	0.015
3. Emotional eating	0.047	−0.188	1	−0.106	**0.691**	−0.096	**0.388**	0.146	0.101	0.193	0.146
4. Cognitive restriction	**−0.280**	−0.059	−0.106	1	−0.129	−0.015	−0.196	−0.131	0.132	0.114	−0.136
5. Uncontrolled eating	0.101	−0.079	**0.691**	−0.129	1	−0.032	**0.329**	**0.258**	−0.083	0.067	0.081
6. Self-esteem	−0.143	−0.075	−0.096	−0.015	−0.032	1	−0.096	−0.241	0.063	0.187	−0.046
7. Anxiety	0.005	0.162	**0.388**	−0.196	**0.329**	−0.096	1	**0.299**	0.040	0.054	−0.075
8. Perceived stress	0.122	0.051	0.146	−0.131	**0.258**	−0.241	**0.299**	1	0.160	−0.038	0.149
9. Engagement *	0.046	−0.097	0.101	0.132	−0.083	0.063	0.040	0.160	1	**0.350**	**0.586**
10. Live sessions	−0.191	**−0.286**	0.193	0.114	0.067	0.187	0.054	−0.038	**0.350**	1	**0.275**
11. Direct messages	0.041	0.015	0.146	−0.136	0.081	−0.046	−0.075	0.149	**0.586**	**0.275**	1

The numbers in bold are statistically significant (*p* < 0.05). Parameters numbered 1 to 8 represent the variation between baseline and post-5-week intervention values. Parameters numbered 9 to 11 represent descriptive values for each participant who completed the 5-week intervention. * Sum of comments and likes on feed posts for each participant.

## Data Availability

Data are contained within the article. The original contributions presented in the study are included in the article/Supplementary Materials; further inquiries can be directed to the corresponding author.

## Supplementary Materials

The following supporting information can be downloaded at https://www.mdpi.com/article/10.3390/nu16234045/s1: Table S1: Pre-protocol Posts and Topics Addressed; Table S2: Week 1 Posts and Topics Addressed; Table S3: Week 2 Posts and Topics Addressed; Table S4: Week 3 Posts and Topics Addressed; Table S5: Week 4 Posts and Topics Addressed; Table S6: Week 5 Posts and Topics Addressed; Live Script S1: Week 1—Habits, Goals, and Readiness for Change; Live Script S2: Week 2—Stress, Emotional Eating, and Psychological Traps; Live Script S3: Week 3—Nutritional Aspects and Number of Meals; Live Script S4: Week 4—Routine Organization and Time Management; Live Script S5: Week 4—Meal Planning, Grocery Shopping, and Food Preparation; Live Script S6: Week 5—Dysfunctional Thoughts that Hinder Weight Loss; Live Script S7: Week 5—The Process Isn’t Over Yet.
