# Supplementary material for: Online Cognitive-Behavioral Therapy-Based Nutritional Intervention via Instagram for Overweight and Obesity
Source: Nutrients. 2024 Nov 26;16(23):4045. doi: 10.3390/nu16234045 (PMC11643239; doi:10.3390/nu16234045)
Supplement: Supplementary file 1 [file nutrients-16-04045-s001.zip › nutrients-3239084-supplementary.pdf]

Supplementary information

Table S1. Pre-protocol Posts and Topics Addressed

Post Title	Topic	Post Type
What is Emagrece.com?	Announcements/ Lives	Photo
Who are we?	Announcements/ Lives	Carousel
Are you ready for change?	Readiness for Change	Carousel

Table S2. Week 1 Posts and Topics Addressed

Post Title	Topic	Post Type
Week 1: Fundamental Strategies	Announcements/Live	Photo
Promotion of the 1st live class	Announcements/Live	Photo
Live Class 1: Habits, Goals, and Fundamental Strategies - Guest Prof. Dr. Brunna Boaventura	Announcements/Live	Live
List of Advantages: Use Them to Your Benefit	Fundamental Strategies	Carousel
Live Summary: Why Are We Here?	Habits and Goals	Carousel
How Habits Are Formed	Habits and Goals	Carousel
Habit Architecture and Stacking	Habits and Goals	Carousel
SMART Goals	Habits and Goals	Carousel
Memories Worth Remembering: An Ace Up Your Sleeve	Fundamental Strategies	Photo

Resistance Muscles	Fundamental Strategies	Photo
Stop Procrastinating	Fundamental Strategies	Photo
"Readiness for Change": How to Move from Contemplation to Action?	Readiness for Change	Reels

Table S3. Week 2 Posts and Topics Addressed

Post Title	Topic	Post Type
Week 2: Food and Emotions	Announcements/Live	Photo
Promotion of the 2nd live class	Announcements/Live	Photo
"Oops, I Overindulged, I'll Keep Allowing Myself..."	Sabotaging Thoughts/ Traps	Photo
Why Do We Eat?	Fundamental Strategies	Carousel
Live Class 2: Stress, Emotional Eating, and Psychological Traps - Guest Prof. Dr. Luciana Antunes	Announcements/ Lives	Live
Live Summary: Stress, Emotional Eating, and Psychological Traps	Sabotaging Thoughts/ Traps	Carousel
Distraction List	Fundamental Strategies	Carousel
"My Day Was Really Tough, I Deserve to Eat Whatever I Want"	Sabotaging Thoughts/ Traps	Photo
Do You Have an Escape Plan?	Fundamental Strategies	Carousel
"My Problem Is Snacking"	Sabotaging Thoughts/ Traps	Carousel

Table S4. Week 3 Posts and Topics Addressed

Post Title	Topic	Post Type
Week 3: Building Healthy Meals	Announcements/Live	Photo
Promotion of the 3rd live class	Announcements/Live	Photo
Live Class 3: Nutritional Aspects and Healthy Meals - Guest Prof. Dr. Brunna Boaventura	Announcements/ Lives	Live
How to Make a Good Breakfast?	Meal Preparation and Food Choices	Carousel
Live Summary: Nutritional Aspects and Healthy Meals	Meal Preparation and Food Choices	Carousel
Building a Good Plate	Meal Preparation and Food Choices	Carousel
How to Choose a Good Snack	Meal Preparation and Food Choices	Carousel
Mindful Eating	Fundamental Strategies	Carousel
Do You Know How to Read Labels?	Meal Preparation and Food Choices	Carousel
Bonus Post: Brazilian Dietary Guidelines (2014)	Meal Preparation and Food Choices	Photo
Week Challenge: A Day Without Ultra-Processed Foods	Meal Preparation and Food Choices	Carousel

Table S5. Week 4 Posts and Topics Addressed

Post Title	Topic	Post Type
Week 4: Organization and	Announcements/Live	Photo

Planning		
Promotion of Bonus Live Class	Announcements/Live	Photo
Are You Putting Yourself First?	Prioritization/ Time Management	Live
Bonus Live Class: Routine Organization and Task Management - Guest Yasmin Bortolotti	Announcements/ Lives	Carousel
Task of the Week: Let's Organize Life?	Prioritization/ Time Management	Carousel
Bonus Meal Plan	Announcements/ Lives	Carousel
Live Summary: Routine Organization and Task Management	Prioritization/ Time Management	Carousel
Promotion of the 5th live class	Announcements/ Lives	Carousel
The Wheel of Life Can Help You!	Prioritization/ Time Management	Carousel
Good Reasons to Cook	Meal Preparation and Food Choices	Photo
Live Class 5: Menu Planning, Organization, and Shopping List - Guest Prof. Dr. Ana Paula Geraldo	Announcements/ Lives	Carousel
Zucchini: 3 Different Versions	Meal Preparation and Food Choices	Photo
Live Summary: Menu Planning, Organization, and Shopping List	Meal Preparation and Food Choices	Carousel

Week Challenge: Plan and Organize the Week's Menu	Prioritization/ Time Management	Carousel

Table S6. Week 5 Posts and Topics Addressed

Post Title	Topic	Post Type
Week 5: Dysfunctional Thoughts and Breaking Routine	Announcements/Live	Photo
Promotion of the 6th live class	Announcements/Live	Photo
Notice: Last Week! Are You Behind on Content? Take This Time to Catch Up	Announcements/ Lives	Photo
Live Class: Dysfunctional Thoughts and Breaking Routine - Guest Psychologist Juliana Bertoletti	Announcements/ Lives	Live
Live Summary: Dysfunctional Thoughts that Hinder Weight Loss	Sabotaging Thoughts/ Traps	Carousel
Just One More Bite" - The Insistence of Others	Sabotaging Thoughts/ Traps	Carousel
Promotion of Bonus Live Class	Announcements/ Lives	Photo
"Will This Be My Last Christmas?"	Sabotaging Thoughts/ Traps	Carousel
Bonus Live Class: Closing - The Process Isn't Over Yet - Guest Prof. Dr. Brunna Boaventura	Announcements/ Lives	Live
Live Summary: The Process	Fundamental Strategies	Carousel

Isn't Over Yet		
"I Overeat During Special Occasions, What Can I Do?"	Sabotaging Thoughts/ Traps	Carousel
Final Questionnaires - Deadline 12/31	Announcements/ Lives	Photo

Source: prepared by the author (2023)

Live Script S1: Week 1 – Habits, Goals, and Readiness for Change

- **Introduction:**
  Discussion on the complexity of weight loss and the importance of readiness for change. Explanation of the 5 stages of readiness for change: pre-contemplation, contemplation, preparation, action, and maintenance, with practical examples.
- **Habit Change:**
  Analysis of the habit structure: cue, craving, response, and reward. Guidance on identifying and modifying habits, focusing on changing the environment to facilitate good choices and hinder undesirable ones.
- **Motivation and Practical Tools:**
  Importance of motivation in the weight loss process. Introduction of the advantages list as a tool to maintain motivation, with examples of advantages and recommendations for daily practice.
- **Fundamental Strategies:**
  Emphasis on self-praise and recognition of personal achievements. Concept of "resistance muscle" versus "giving up muscle" and strategies to strengthen resistance to temptation.

- **Conclusion:**
  Summary of discussions and tasks for the week, such as creating an advantages list and sharing it on social media stories.

Live Script S2: Week 2 – Stress, Emotional Eating, and Psychological Traps

Introduction: The session covers the interaction between stress, emotional eating, and psychological traps that hinder weight management.

Emotional Eating: Exploration of emotional eating as a response to negative emotions such as stress, sadness, and frustration. Discussion on how food is often used to numb emotions rather than addressing them directly, leading to dysfunctional eating behaviors.

Psychological Traps: Identification of psychological traps, such as sabotaging thoughts and resistance to change, that can lead to dysfunctional responses to emotions. Emphasis on the need to recognize these patterns and replace them with adaptive, goal-oriented behaviors.

Cognitive Strategies: Introduction of techniques to combat sabotaging thoughts, including the development of confronting thoughts and adaptive responses. Practical examples are provided to illustrate how these strategies can be applied in everyday situations.

Conclusion: Summary of key points, including the importance of recognizing emotional triggers, identifying sabotaging thoughts, and applying adaptive responses. Encouragement to develop a personalized action plan to address psychological traps and enhance the success of weight management efforts.

Live Script S3: Week 3 – Nutritional Aspects and Number of Meals

Introduction: The session begins with an exploration of foundational concepts in nutrition and the importance of meal frequency in the context of weight loss. The introduction highlights that, while the primary focus is on behavior, understanding nutritional aspects is crucial for effective weight management.

Calories and Energy Balance: Explanation of what calories are and their role in providing energy for the body's vital functions and daily activities. The discussion covers the concepts of caloric intake and expenditure, emphasizing the importance of maintaining a caloric balance for weight management. The concept of caloric surplus and deficit is explained, stressing that no single food inherently causes weight gain or loss—context and total caloric intake are key.

Caloric Density: Introduction to the concept of caloric density, which refers to the number of calories per gram of food. Examples are provided to illustrate how foods with high caloric density (like condensed milk) can contribute to excessive caloric intake, while foods with low caloric density (like fruits and vegetables) promote satiety without adding many calories.

Meal Frequency: Discussion on the number of meals one should have in a day, with an emphasis on the benefits of meal fractionation for weight loss. It is explained that spreading meals throughout the day can help manage hunger levels and prevent overeating during specific meals. The importance of paying attention to hunger cues and avoiding excessive snacking is also highlighted.

Macronutrients and Micronutrients: Overview of essential macronutrients—carbohydrates, proteins, and lipids—and their roles in the body. Practical advice is given on how to incorporate these macronutrients into daily meals for a balanced diet that supports weight loss. Additionally, the role of micronutrients (vitamins and minerals) in supporting metabolic functions is briefly discussed.

Food Classification and Label Reading: Introduction to the Brazilian Food Guide's classification of foods into in natura, minimally processed, processed, and ultra-processed categories. Emphasis is placed on the importance of basing the diet on in natura and minimally processed foods while limiting processed and avoiding ultra-processed foods. Practical guidance is provided on how to read food labels, with tips on understanding nutritional information and ingredient lists to make informed food choices.

Common Pitfalls: Discussion of common pitfalls in eating habits, such as eating too quickly, repeating meals without hunger, and being distracted during meals. Strategies are suggested to avoid these pitfalls, such as waiting before taking a second serving, prioritizing the repetition of vegetables and proteins, and minimizing distractions during meals.

Conclusion: The session concludes with a summary of the key points discussed and an encouragement for participants to engage in self-monitoring by recording their meals for three days. This exercise is intended to increase awareness of eating patterns and caloric intake, aiding in the development of healthier habits.

Live Script S4: Week 4 – Routine Organization and Time Management

Introduction: Importance of organizing both diet and life to manage responsibilities and achieve goals.

Prioritization and Management: Identify and prioritize important areas, maintaining discipline and adapting to needs.

Setting Priorities: Establish goals based on real desires and prioritize self-care.

Importance of Routine: Improves mental health, reduces stress, and increases productivity.

Creating a Routine: Integrate new habits with existing ones, prepare materials in advance, be consistent yet flexible with the routine.

Procrastination: Understand causes and strategies to overcome it.

Practical Tips: List tasks, estimate times, schedule by periods, and include breaks.

Conclusion: Summary and encouragement to apply the discussed strategies.

Live Script S5: Week 4 – Meal Planning, Grocery Shopping, and Food Preparation

Introduction: Exploration of the importance of meal planning, organization, and grocery shopping for maintaining a healthy diet. Emphasis on how effective planning prevents impulsive eating decisions and helps achieve dietary goals.

Meal Planning: Steps for organizing a weekly menu, including:

1. Assessing current food inventory by checking the pantry, freezer, and fridge.
2. Listing potential meals based on available ingredients and realistic expectations.
3. Creating a shopping list for missing items and planning grocery trips.
4. Establishing a weekly shopping day and avoiding shopping on an empty stomach to reduce impulse buys.

Food Preparation and Organization: Guidance on efficient food preparation:

1. Immediate cleaning and storage of groceries upon return from shopping.
2. Prepping fruits and vegetables for easier access during the week.
3. Cooking larger quantities and freezing portions for convenience.
4. Utilizing strategies like pre-cutting fruits and storing greens properly to extend shelf life.

Family Considerations: Tips for accommodating various family preferences and managing diverse tastes:

1. Identifying common foods that can be adapted for different preferences.
2. Creating a family meal plan that balances individual likes and nutritional needs.
3. Being flexible with food choices and making substitutions as needed.

Conclusion: Summary of key points and encouragement to implement these strategies. Reminder to experiment with meal planning and preparation techniques to find what works best for individual needs and family dynamics.

Live Script S6: Week 5 – Dysfunctional Thoughts that Hinder Weight Loss

Introduction: Exploration of the challenges faced during festivities and moments that disrupt routine, and how the influence of family and friends can impact the weight loss process. Discussion on how to stay focused amidst these situations and the importance of handling external pressure.

Influence Traps:

1. **Pressure to Eat:h**ow insistence from family members, such as grandparents offering food, can lead to undesired acceptance;recommended strategy: Broken Record Technique, which involves repeating "No, thank you" until the insistence stops; importance of not feeling obliged to justify refusals and maintaining firmness in your dietary decisions.
2. **Influence and Acceptance:** difficulties in saying no due to fear of disappointing others, compared to choosing veganism or vegetarianism, need to prioritize your own needs and goals over others' expectations.

Dealing with Others' Reactions:

1. **Concerns about Family and Friends' Reactions: e**valuating the consequences of refusing food versus giving in, reflection on the personal costs of giving in, such as impact on weight loss and self-frustration.
2. **Judgment and Resistance:** how to handle judgments and negative comments from family and friends, recognizing that criticisms may arise from insecurities and resistance to changes in others.

Self-Sabotage and Flexibility:

1. **Personal Sabotage:**
   - Challenges in maintaining routine and dealing with dietary mistakes, importance of quickly returning to routine after a deviation, avoiding the "all or nothing" mindset.
2. **Anxiety and Festivities:**
   - Impact of anxiety on dietary decisions and how to manage it, strategies for maintaining control during holidays and avoiding excesses based on the "you only live once" mindset.

Strategies for Festivities:

1. **Preparation and Planning:** Creating a list of advantages for festivities and the importance of early planning, maintaining regular meals and avoiding skipping meals before events.
2. **Behavior and Food Choices:** Eating slowly and paying attention to the food, selecting foods critically and setting goals for portion sizes.

Conclusion: Summary of key points discussed and encouragement to apply the proposed strategies. Encouragement to reflect on the importance of staying focused and prioritizing personal goals during festivities and social situations.

Live Script S7: Week 5 - The Process Isn't Over Yet

Introduction:

- **Review of Key Points:** Discussion on the essential elements of the protocol and the importance of continuing to prioritize yourself even as the program concludes. Emphasis on not waiting for others to change and maintaining personal commitment.

Maintaining Focus:

- **Self-Prioritization:** Reiterate the need to make room for your goals in your schedule. The actions and commitment must come from you, and you should not rely on external changes.
- **Advantages List:** Reminder of the advantages list introduced in Week 1 as a vital tool for maintaining motivation and focus.

Identifying and Addressing Sabotaging Thoughts:

- **Understanding Triggers:** Overview of how to identify sabotaging thoughts related to eating due to emotions, stress, boredom, or external pressures.
- **Escape Plan Strategy:** Strategies for countering sabotaging thoughts and staying on track. Emphasize creating confrontational thoughts to reinforce your commitment to the plan.

Key Lessons and Daily Choices:

- **Returning to the Path:** Highlight the critical lesson that the main error is not returning to your plan after a deviation. Stress the importance of getting back on track quickly.
- **Daily Choices:** Importance of making the best choices daily, selecting your battles wisely, and reducing harm where possible.

Recap of Key Topics:

- Briefly summarize all topics covered throughout the protocol and the strategies discussed in previous weeks.

Q&A and Next Steps:

- **Questions:** Address questions about continuing independently, how long to follow the meal plan, and maintaining lost weight.
- **Final Protocol Steps:** Guidance on concluding the protocol, including final questionnaires and next steps.
